# 910. Herpes Zoster in Children: A Literature Review

**DOI:** 10.1093/ofid/ofad500.955

**Published:** 2023-11-27

**Authors:** Chris Simpson, Anne Dilley, Emma Viscidi

**Affiliations:** Epidemiologic Research & Methods, LLC, Southport, North Carolina; Epidemiologic Research & Methods, LLC, Southport, North Carolina; Moderna Therapeutics, Cambridge, Massachusetts

## Abstract

**Background:**

Herpes zoster (HZ), known as shingles, is caused by reactivation of the varicella zoster virus (VZV) and is characterized by a painful and/or itchy vesicular rash. The incidence of HZ in adults is approximately 4-10 per 1,000 person-years (PY). While the condition occurs mostly in older adults, it can develop in childhood after primary infection or rarely varicella vaccination (VV). The contemporary burden of HZ in children, and the impact of VV policies on incidence rates (IRs), are not well described.

**Methods:**

A systematic literature search was conducted to identify studies reporting the incidence of HZ among pediatric populations (< 20 or < 18 years of age). A search of peer-reviewed literature published from 2010 onward in the PubMed and EMBASE databases was conducted of studies published through January 31st, 2023. While reviewing potentially relevant papers, reference lists were explored to discover additional peer-reviewed articles and reports. Publications were eligible for inclusion if they reported estimates of the IR of medically-attended HZ in nationally-representative samples of pediatric patients, using clearly stated and defensible methods.

**Results:**

Sixteen publications meeting criteria were identified. IRs increased with age, from a median of 0.15 per 1,000 PYs in < 1 year old to 1.88 per 1,000 PYs in 15-19 years of age. In 3 countries with long-standing VV programs, IRs were 0.38 per 1,000 PY in the US, 0.82 per 1,000 PY in Canada, and 1.2 per 1,000 PY in Germany. In countries without widespread VV, incidence was higher, ranging from 0.8 per 1,000 PY in Norway to 2.5 per 1,000 PY in the Netherlands. The median hospitalization rate in children across 5 studies was 2.0 per 100,000 per year (range: 0.4 to 3.7). Immunocompromised (IC) children are at higher risk for HZ; in two studies that compared HZ in IC and non-IC children, the incidence rate ratios were 5.7 and 8.6. Post-herpetic neuralgia, the largest contributor to the HZ health burden, is much less likely to develop in children.

**Conclusion:**

Based on a contemporary literature review, the incidence of HZ was lower in the children than in adults and HZ IRs were lower in countries with routine childhood VV. The incidence in children increased with age and is higher in the immunocompromised.

**Disclosures:**

**Chris Simpson, MSPH**, Epidemiologic Research & Methods: Advisor/Consultant **Anne Dilley, PhD**, Epidemiologic Research & Methods: Advisor/Consultant **Emma Viscidi, PhD, MHS**, Moderna: Employee|Moderna: Stocks/Bonds

